# Experience of a Referral Center with Desmoid Tumors, Part 2: A Retrospective Analysis of 109 Cases

**DOI:** 10.3390/cancers18020305

**Published:** 2026-01-19

**Authors:** Rosa Alvarez Alvarez, Carolina Agra Pujol, Marta Arregui Valles, Francisco Alijo, Adriana Fernández Gonzalo, Natalia Gutiérrez, Pablo Lozano Lominchar, Cristina Mata Fernández, Lydia Mediavilla Santos, Ulrike Novo, Marina Santos, Guillermo Hernández Torrado, Henar Carpintero García, Ana Gutiérrez-Ortiz de la Tabla

**Affiliations:** 1 Medical Oncology Department, Instituto de Investigacion Sanitaria Gregorio Marañon, Hospital General Universitario Gregorio Marañon, 28007 Madrid, Spain; marta.arregui@salud.madrid.org (M.A.V.); ngutierrezalonso@salud.madrid.org (N.G.); ghernandezt@salud.madrid.org (G.H.T.); 2 Pathology Department, Hospital General Universitario Gregorio Marañon, Complutense University, 28007 Madrid, Spain; carolina.agra@salud.madrid.org (C.A.P.); francisco.alijo@salud.madrid.org (F.A.); 3 Radiology Department, Hospital General Universitario Gregorio Marañon, 28007 Madrid, Spain; afgonzalo@salud.madrid.org (A.F.G.); ulrike.novo@salud.madrid.org (U.N.); 4 General Surgery and Surgical Oncology Department, Hospital General Universitario Gregorio Marañon, Complutense University, 28040 Madrid, Spain; pablo.lozano@salud.madrid.org; 5 Gregorio Marañón Materno Infantil Hospital, 28009 Madrid, Spain; cristina.mata@salud.madrid.org; 6 Orthopedic Surgery and Traumatology Department, Musculoskeletal Oncology Section, Hospital General Universitario Gregorio Marañon, Complutense University, 28040 Madrid, Spain; lydia.mediavilla@salud.madrid.org; 7 Radiation Oncology Department, Hospital General Universitario Gregorio Marañon, 28007 Madrid, Spain; marina.santos@salud.madrid.org; 8 Orthopedic Surgery and Traumatology Department, Hospital General Universitario Gregorio Marañon, 28007 Madrid, Spain; henar.carpintero@gmail.com; 9 Medical Oncology Department, Hospital Universitario Infanta Leonor, 28031 Madrid, Spain

**Keywords:** desmoid-type fibromatosis, intra-abdominal desmoids, extra-abdominal desmoids, familial adenomatous polyposis (FAP), *CTNNB1* mutation, active surveillance, multidisciplinary management, referral center experience, desmoid tumor

## Abstract

Background: Desmoid tumors (DTs) are rare, locally aggressive fibroblastic neoplasms with highly heterogeneous clinical behavior. The present work constitutes the second part of a two-part project, following our previously published multidisciplinary review of the diagnostic and therapeutic landscape of DTs. It provides a comprehensive analysis of our institutional experience as a national reference center for sarcoma. We aim to describe real-world diagnostic pathways, management strategies, and clinical outcomes in a high-volume cohort. Methods: We conducted a retrospective cohort study that included patients diagnosed with DT at our center between 2014 and 2024. Demographic, clinical, molecular, treatment, and outcome data were collected. Management strategies were analyzed according to tumor location, symptoms, progression patterns, and multidisciplinary decision-making. Outcomes included response rates, event-free survival (EFS), need for active treatment, response to systemic therapy, and recurrence after local treatments. Results: A total of 109 patients were included (median age 36.8 years; 56.9% women). Somatic *CTNNB1* mutations were identified in 23 of 29 tested patients, predominantly *T41A*, while germline alterations were found in 18 patients, mainly in *APC*. Initial management was conservative in 40.4% of patients and active in 59.6%, primarily through surgery. After a median follow-up of 41.5 months, 44.9% of patients experienced disease progression. Among patients managed with active surveillance, spontaneous regression occurred in 22.2%, and 58% remained treatment-free. Surgical relapse occurred in 35.8% of patients undergoing upfront resection, with major postoperative complications limited to externally operated cases. Cryoablation achieved radiological responses in most evaluable patients, while systemic therapies showed clinical activity but relevant toxicity, particularly with tyrosine kinase inhibitors. The median EFS for the whole cohort was 57 months. Conservative initial management and R1/2 surgical margins were independently associated with worse EFS. Conclusions: Our results support a personalized, multidisciplinary management strategy for DTs, prioritizing conservative approaches when appropriate and reserving active treatments for progressive or symptomatic disease. Outcomes achieved in a specialized referral center are comparable to those reported in large international retrospective series, underscoring the value of expert multidisciplinary care in optimizing DT management.

## Simple Summary

Desmoid tumors are rare soft tissue neoplasms with unpredictable behavior. Although they do not metastasize, they can grow locally and cause pain or functional impairment. Due to the potential for spontaneous regression, therapeutic strategies have recently evolved toward more conservative and individualized approaches, prioritizing active surveillance and functional preservation whenever possible. This study presents the experience of our national sarcoma reference center, reviewing more than 100 patients treated over the past decade. We analyze demographic, clinical, radiological, and treatment-related variables to explore potential prognostic factors associated with disease evolution and therapeutic outcomes. Our findings contribute to a better understanding of desmoid tumor behavior in a real-world, multidisciplinary setting, which will help support more individualized decision-making in routine clinical practice.

## 1. Introduction

Desmoid tumors are rare, locally aggressive soft tissue neoplasms classified by the World Health Organization (WHO) as intermediate-malignancy tumors due to their infiltrative growth and lack of metastatic potential [1,2].

These tumors arise from a monoclonal proliferation of fibroblasts and myofibroblasts and can occur in virtually any anatomical location, affecting both children and adults, with an estimated incidence of less than five cases per million per year [1,2,3]. Most sporadic tumors (>85%) harbor somatic *CTNNB1* mutations, whereas 5–10% are associated with germline *APC* variants within the context of familial adenomatous polyposis (FAP) [1,2,4,5]. These alterations converge in aberrant activation of the Wnt/β-catenin pathway, recognized as the main molecular driver of DTs. The clinical course of DTs is highly heterogeneous, ranging from spontaneous stabilization or regression—reported in up to 20–50% of cases—to progressive local invasion that may result in pain, functional impairment, or significant morbidity [3,6,7]. This unpredictable behavior has led to a paradigm shift in management, moving away from upfront surgery toward more conservative, patient-centered approaches. The Desmoid Tumor Working Group and other international expert panels now endorse active surveillance (AS) as the initial strategy for asymptomatic or non-life-threatening tumors, reserving intervention for cases with progression, symptomatic burden, or anatomical risk [3,6,7].

When treatment is indicated, options include systemic therapies, local ablative techniques, radiotherapy (RT), and selected surgery, ideally within a multidisciplinary framework [6,7].

Systemic therapy for DTs has expanded considerably in the past decade [8,9]. Traditional regimens such as methotrexate–vinblastine and anthracycline-based chemotherapy (particularly pegylated liposomal doxorubicin), remain valid options, but targeted agents have gained prominence [10,11]. Tyrosine kinase inhibitors (TKIs), including sorafenib and pazopanib, have demonstrated clinically meaningful activity in randomized trials [12,13,14]. More recently, gamma-secretase inhibitors (GSIs), particularly nirogacestat, have shown high efficacy and improved patient-reported outcomes, leading to their regulatory approval for progressive DTs in adults [15,16]. In contrast, HT ± NSAIDs have consistently demonstrated limited or no meaningful clinical benefit and are no longer considered an effective therapeutic approach in contemporary management [7,8,17].

Despite these advances, significant knowledge gaps persist. Prognostic factors capable of predicting the natural history or treatment response are not yet fully established, and evidence regarding the optimal sequencing and duration of systemic therapy remains limited [6,7,17]. The burden of disease—including pain, functional limitation, and psychosocial impact—remains substantial, underscoring the need for ongoing research and collaborative efforts to optimize outcomes and quality of life for patients with DTs.

In this second part of our review, we present a retrospective analysis of more than 100 patients diagnosed and managed at our national sarcoma reference center over the past decade, exploring clinical trajectories, treatment approaches, and potential prognostic factors. These real-world data complement the multidisciplinary recommendations detailed in Part 1 of our study and contribute to more harmonized and precise management strategies for patients with desmoid-type fibromatosis [17].

## 2. Methods

A retrospective cohort study was conducted to evaluate the clinical characteristics and outcomes of patients diagnosed with DTs and managed at a national sarcoma referral center.

### 2.1. Study Population

This study included patients with a confirmed diagnosis of DT between 2014 and 2024. A sarcoma-specialized pathologist at our institution evaluated all available histopathological specimens to establish the diagnosis. In select high-risk biopsy cases where tissue sampling posed a significant clinical risk, the diagnosis was established based on concordant clinical and radiological findings. A dedicated multidisciplinary tumor board evaluated all cases. Three sarcoma-specialized medical oncologists from the center independently reviewed the medical records.

This study received approval from the relevant institutional ethics committee.

### 2.2. Study Outcomes and Variables

The data collected included gender, diagnosis date, tumor size, primary tumor location, molecular alterations, and treatment modalities.

Tumor location was classified into four anatomical categories based on the site of the primary lesion: (1) abdominal wall; (2) intra-abdominal, pelvic, or retroperitoneal; (3) extremities, girdles, or thoracic wall; and (4) head and neck. For tumors involving multiple anatomical regions, classification was based on the predominant site of involvement.

Molecular profiling for somatic mutations in *CTNNB1* or *APC* genes was performed in most patients diagnosed between 2021 and 2024, using a custom gene panel with capture-based enrichment (Custom Solid Tumor Solution, Sophia Genetics, Lausanne, Switzerland), followed by sequencing on a MiSeq platform (Illumina, San Diego, CA, USA). Germline *APC* testing was performed at the discretion of the treating physician based on clinical features or family history suggestive of hereditary syndromes.

Initial therapeutic approaches were categorized as either active or conservative. Active treatments included systemic anti-cancer therapy—such as chemotherapy (CT) or TKIs—surgery with curative intent, and local techniques (cryoablation or RT). Conservative management included hormonal therapy with or without non-steroidal anti-inflammatory drugs (HT ± NSAIDs) and AS. In this study, AS was defined as regular clinical and radiological monitoring, with Magnetic Resonance Imaging (MRI) or Computed Tomography scan (CT scan) imaging performed every 8 to 12 weeks. Surgical margins were classified as negative (R0), microscopically positive (R1, marginal), or macroscopically positive (R2, gross residual disease).

Treatment response was assessed by radiologists specializing in soft tissue tumors using a CT scan or MRI. Responses were classified as complete response (CR), partial response (PR), stable disease (SD), or progressive disease (PD) according to RECIST 1.1 criteria. In the context of AS, tumor behavior was categorized as complete spontaneous regression, partial spontaneous regression, SD, or PD. For patients managed with AS, a study event was defined as a change in therapeutic strategy rather than radiological evidence of progression. EFS was defined as the time from initial diagnosis to the occurrence of the first event, either local recurrence or progression, change in treatment approach, death from any cause, or censoring at the date of the last follow-up. In patients receiving active treatment, EFS was calculated from the start of the first active therapeutic intervention to the occurrence of the first event, defined as disease progression or recurrence, change in treatment, death from any cause, or censoring at the date of last follow-up.

### 2.3. Statistical Analysis

Data were analyzed using SPSS software version 21.0 (IBM SPSS, Chicago, IL, USA). Continuous variables were expressed as medians and interquartile ranges (IQRs), and categorical variables were presented as percentages. Cumulative event rates were estimated using the Kaplan–Meier method, and survival curves were compared using the log-rank test. Prognostic factors associated with EFS were identified through univariate and multivariate analyses using the Cox proportional hazards regression model. All statistical tests were two-sided, with a *p*-value < 0.05 considered statistically significant.

## 3. Results

From 2011 to 2024, a total of 109 patients with DT were included in the analysis. The majority were female (*n* = 62, 56.9%) with a median age at diagnosis of 36.8 years (IQR 29–48). Baseline cohort characteristics by initial treatment strategy (active vs. conservative) are shown in Table 1.

Somatic *CTNNB1* gene mutations were assessed in 29 patients, of whom 23 (21.1% of the overall cohort) harbored a pathogenic variant. The most common mutation was T41A (*n* = 17, 58.6%), followed by *S45F* (*n* = 3, 10.3%) and *S45P* (*n* = 3, 10.3%). Germline testing was performed in 40 patients, revealing *APC* mutations in 18 patients (16.5% of the total cohort). Additionally, isolated germline mutations in *POT1*, *MUTYH*, and *BRCA1* were detected in one patient each (Table 2).

Of the entire cohort, 65 patients (59.6%) received active front-line treatment, which included surgery (*n* = 54, 49.5%), cryoablation (*n* = 4, 3.7%), RT (*n* = 1, 0.9%), or systemic therapy with CT or TKIs (*n* = 6, 5.5%). The remaining 44 patients (40.4%) were managed with conservative approaches, consisting of HT with or without NSAIDs (*n* = 20, 18.4%) and AS (*n* = 24, 22.0%).

During a median follow-up of 41.5 months, radiologic PD occurred in 49 patients (44.9%) under the initial therapeutic approach. Among them, 33 patients received second-line therapeutic interventions, including surgery (*n* = 18), CT/TKIs (*n* = 8), cryoablation (*n* = 6), and RT (*n* = 1). The remaining patients were managed conservatively: 7 with AS and 9 with HT ± NSAIDs.

### 3.1. Active Surveillance

Among the 36 patients who underwent AS during their disease course, 8 spontaneous regressions were reported (22.2%), including 2 cases (5.5%) of complete tumor regression; 19 patients (52.8%) had SD as the best response, while 9 patients (25%) presented PD as the best response.

A total of 24 patients (22.0% of the cohort) were managed with AS as the initial treatment strategy at the time of diagnosis (37.5% intra-abdominal, 37.5% extremities, and 25% abdominal wall) (Table 1). Among these 24 patients, 14 (58.3%; 12.8% of the overall population) remained untreated and experienced no tumor-related events after a median follow-up of 22.5 months (IQR 7.9–49.5). The remaining 10 patients switched to an active treatment after a median observation period of 4.5 months (IQR 3.0–18.0), primarily due to repeated progression (*n* = 9) or, in one case, risk of bowel obstruction.

An additional 12 patients initially received other treatments (surgery, *n* = 8; HT, *n* = 2; cryoablation, *n* = 2) and subsequently underwent AS as a second (*n* = 7) or later line (*n* = 5) of treatment following progression or relapse (Table 3). Notably, 8 of these patients (66.7%) remained progression-free without further intervention during the follow-up.

### 3.2. Hormone Therapy ± NSAIDs

A total of 32 patients received HT with tamoxifen, either alone or in combination with non-steroidal anti-inflammatory drugs, including celecoxib, sulindac, or indomethacin.

Twenty patients (18.3% of the overall population) received HT as the initial strategy. The characteristics of this subgroup are detailed in Table 1. Of note, most patients presenting with multifocal disease received HT *±* NSAIDs as the first approach (*n* = 17/24, 70.8%).

Most patients showed SD as the best response (*n* = 12, 60%), 2 patients (10%) experienced tumor regression, and PD was the best response in 4 patients (20%).

Of the 20 patients (30%) with favorable responses, 6 required no further treatment, whereas the remaining 14 experienced PD or another event after a median follow-up of 55.4 months (37.5–89.3), with a median EFS of 35.5 months [95% CI 7.3–68.3].

### 3.3. Surgery

A total of 67 patients (61.5%) underwent surgery at some point during their disease course. Among the patients who underwent surgery, the most common primary tumor site was the extremities/girdles/thoracic wall (*n* = 26, 38.8%), followed by intra-abdominal/retroperitoneum/pelvis (*n* = 24, 35.8%), abdominal wall (*n* = 12, 17.9%), and head and neck (*n* = 5, 7.5%). Eleven patients (16.4%) had a tumor involving multiple sites (Table 3).

Reported indications for surgery included tumor location threatening vital structures (*n* = 11, 16.4%), uncontrollable pain (*n* = 6, 9%), PD after AS (*n* = 3, 4.5%), long-standing symptomatic progression before diagnosis (*n* = 2, 3%), and incidental diagnosis (*n* = 6, 9%). In the remaining 39 cases (58.2%), no specific indication for surgery was reported. Information on surgical margins was available for 62 patients: R0 in 40 cases (64.5%), R1 in 18 (29.0%), and R2 in 4 (6.5%). Of these, 6 patients received complementary treatments, either adjuvant HT ± NSAIDs (*n* = 4; 2 with R2 margins, 1 with R1, and 1 with R0) or adjuvant RT (*n* = 2; one with R1 margins and one with unknown margin status).

Fifty-four patients (49.5%) received surgery as an initial treatment approach, and their characteristics are detailed in Table 1. Tumor relapse occurred in 24 patients (35.8%) after initial surgeries (11 of which were performed at external institutions), with a median relapse-free survival of 26.5 months (IQR 3.1–87.0). Salvage surgery was performed in 8 of these patients (33.3% of relapsed patients), all of whom were referred from other centers. Other strategies undertaken after relapse were HT ± NSAIDs (*n* = 7, 29.2%), AS (*n* = 6, 25.0%), CT/TKIs (*n* = 2), and RT (*n* = 1).

Twelve patients received surgery following failure of prior therapies (7 after HT ± NSAIDs, 3 after AS, and 2 after undergoing more than one prior strategy), and 1 patient received surgery after neoadjuvant CT.

Reported severe postoperative events included an anatomical fistula in two patients and severe short bowel syndrome in one patient, which ultimately resulted in death due to related complications. Of note, all these patients had surgery outside of our network.

### 3.4. Cryoablation

Cryoablation was delivered at all tumor sites except intra-abdominal locations (Table 3). The most frequent tumor location was the extremities/girdles/thoracic wall (*n* = 9, 69.2%). Among the 13 patients that underwent cryoablation, 12 were evaluable for response, among whom 9 (69.2%) showed a radiological response—including 2 CRs—and did not progress during the follow-up. Of the 3 patients who did not respond, 2 underwent a second ablative procedure after 3 months and also had PD as the best response. Both patients responded to subsequent CT.

Only 4 patients received cryoablation as the initial strategy, and 1 event occurred (PD after repeat cryoablation) after a median follow-up period of 16.4 months (IQR 6.3–35.0).

In the remaining cases, cryoablation was administered after failure of previous treatments: HT ± NSAIDs (*n* = 4), AS (*n* = 2), and surgery (*n* = 3).

### 3.5. Radiation Therapy

RT was administered to 10 patients in the cohort (9.2%). Only 1 patient—who had a head and neck DT—received RT as the initial strategy (details shown in Table 1), receiving a total dose of 60 Gy and achieving a maintained PR, with no evidence of relapse after a follow-up of 145 months. In 2 cases, RT was administered postoperatively (R1 resection in 1 case, 50 Gy; margin status unknown in the other, 56 Gy). Two patients received intraoperative RT (Table 3).

No grade 3–4 toxicities were reported in association with RT. However, 1 patient developed a grade 2 undifferentiated spindle cell sarcoma at the site of a previous DT in the buttock, which had been treated with surgery and postoperative RT 6 years before. This was considered a potential radiation-induced second malignancy. Germline analysis revealed a *POT1* gene mutation in this patient.

### 3.6. Anti-Cancer Medical Treatment (CT/TKIs)

Twenty-six patients (23.9%) received at least one line of CT/TKIs.

Of the total population, anti-cancer medical treatment was the initial strategy used in only 6 patients (5.5%) (Table 1). Four events occurred during a median follow-up of 16.8 months (IQR 6.1–55.4), with a median EFS of 8 months (95% CI 0.5–15.5).

CT/TKIs were used as a second-line approach in another 6 patients: 2 patients following relapse after surgery, 2 after progression on cryoablation, and 2 after failure of HT ± NSAIDs. The remaining 8 patients received CT/TKIs as a subsequent treatment after the failure of 2 or more strategies.

The most frequently used agents and their respective response rates are shown in Table 4. Other drugs administered were pazopanib (*n* = 2), imatinib (*n* = 1), sunitinib (*n* = 2), and taxanes (*n* = 2).

#### 3.6.1. Toxicity

##### Tyrosine Kinase Inhibitors

Grade 3–4 adverse events were mainly observed in patients treated with TKIs. Among patients receiving sorafenib, grade 3–4 toxicities occurred in 5 patients (38.5%), including dermatologic toxicity (*n* = 2), hepatotoxicity (*n* = 2), and thrombocytopenia (*n* = 1). Among patients treated with pazopanib, 1 patient discontinued therapy due to grade 3 febrile neutropenia and anemia. No additional grade ≥ 3 events reported.

No grade 5 toxicities were reported in patients receiving TKIs.

##### Chemotherapy

Chemotherapy was generally well tolerated. The only grade 3 adverse event observed was peripheral neuropathy in 1 patient treated with methotrexate and vinblastine. No grade 4 or 5 toxicities were reported in patients receiving CT.

### 3.7. Results for Specific Populations

#### 3.7.1. Pregnancy and DT

Five patients in the cohort were pregnant at some point during their disease course (*n* = 2 abdominal wall, *n* = 1 head and neck, and *n* = 2 extremities/girdles). One patient maintained an SD during pregnancy, whereas 4 had PD, including 1 patient with a previously resected abdominal wall DT who experienced relapse during pregnancy.

Three patients received conservative management following delivery. Two underwent AS and experienced tumor regression (1CR and 1 PR), with no further relapse reported after a median follow-up of 29.5 months. One patient was treated with HT ± NSAIDs, achieving sustained SD over 36 months of follow-up.

Two patients were started on active therapies due to rapid progression after delivery. One patient with an extremity DT underwent R1 resection and relapsed after 12 months. Salvage R0 surgery was performed, with no subsequent recurrence during 31 months of follow-up. The other patient, with PD, received systemic treatment with liposomal doxorubicin and achieved a sustained PR after starting second-line sorafenib, with no further progression reported after 26 months.

#### 3.7.2. Childhood DTs

Eight patients were aged ≤ 18 years at the time of DT diagnosis (median age 16; range: 2–18). Five patients (62.5%) were male, and 3 (37.5%) were female. The most common tumor location was the extremities (*n* = 4, 50%), followed by the intra-abdominal region (*n* = 2, 25%), with 1 patient each presenting tumors in the abdominal wall and head and neck.

Somatic *CTNNB1* mutation testing was performed in 3 patients, all of whom revealed *T41A* mutations. Germline *APC* testing was conducted in 4 patients, 2 of whom tested positive. One patient had previously undergone FAP-related surgery. The initial treatment strategies for children were surgery (*n* = 3, 37.5%), AS (*n* = 2, 25%), HT ± NSAIDs (*n* = 2, 25%), cryoablation (*n* = 1, 8.7%), and CT (*n* = 1, 8.7%).

#### 3.7.3. DT and History of Prior Trauma

A history of local trauma before DT diagnosis, except for previous surgical interventions, was scarcely recorded in the medical charts. In total, 24 patients (22.0%) developed a DT at the site of a previous surgery. Fourteen of these cases received prophylactic surgery for FAP. Among the remaining 10, prior surgeries included resection of other tumors (*n* = 4) and non-oncologic procedures (*n* = 6). Only 4 of these patients underwent germline *APC* testing, with only one testing positive.

### 3.8. Study Outcomes

#### 3.8.1. Event-Free Survival

The median follow-up was 41.5 months. During this period, 51 events were recorded. The median EFS for the whole population was 57 months (95% CI 32.4–81.6).

Kaplan–Meier analysis did not show statistical differences according to the choice of initial treatment strategy (active vs. conservative; log-rank *p* = 0.168).

#### 3.8.2. Predictors of Event Free Survival

In the univariate analysis using Cox proportional hazards, sex (*p* = 0.86), primary tumor site (*p* = 0.55), *CTNNB1* mutation (presence vs. absence, *p* = 0.10; type of mutation, *p* = 0.96), choice of first treatment (*p* = 0.17), and multiple anatomical sites (*p* = 0.13) did not influence EFS. The presence of the *APC* gene mutation (*p* = 0.02) and symptoms at diagnosis (*p* = 0.02) were associated with worse EFS in univariate analysis. Age (*p* = 0.06), tumor size (<5 vs. >5 cm) (*p* = 0.08), and surgical margin status (*p* = 0.07) showed a trend towards statistical significance.

A multivariable Cox regression model was built, including variables that were significant in univariate analysis and those deemed clinically relevant. In this model, initial treatment with conservative strategies (vs. active therapy) was independently associated with a higher risk of events (HR = 5.36; 95% CI: 1.95–14.78; *p* = 0.001), as was a R1/R2 surgical margin status (HR = 2.26; 95% CI: 1.26–4.05; *p* = 0.006). Tumor size ≥ 5 cm showed a non-significant trend toward worse EFS (HR = 2.00; 95% CI: 0.88–4.56; *p* = 0.100).

#### 3.8.3. Overall Survival

Median overall survival for the cohort was not reached. At the time of data cutoff, 105 patients (96.3%) were alive. There were 4 deaths: one due to FAP-associated desmoid tumor, 1 related to post-surgical complications, and 2 from causes unrelated to the disease.

## 4. Discussion

This retrospective cohort study provides a comprehensive overview of the clinical characteristics, molecular features, treatment strategies, and outcomes of more than 100 patients with DTs managed at a national referral center over a ten-year period. Our findings reflect real-world clinical practice and underscore the complexity and heterogeneity of DTs, both in terms of biological behavior and therapeutic decision-making, and highlight the value of a multidisciplinary approach within a specialized referral setting. It is worth noting that our cohort may include a higher proportion of complex, aggressive, or previously poorly managed cases referred from other centers. This selection bias may distort the observed need for active intervention and overestimate recurrence rates (e.g., after external surgery); therefore, we must exercise caution when generalizing the results. Consistent with previous studies in the literature, most patients in our cohort were young adults and female, with extremities and intra-abdominal regions being the most frequent tumor sites [3,7]. The integration of molecular profiling in recent years has enhanced diagnostic precision and may have prognostic implications, particularly concerning *CTNNB1* and *APC* mutations [2,4,7]. Notably, a relevant proportion of patients harbored germline *APC* alterations, underscoring the importance of considering hereditary syndromes in selected cases, particularly in pediatric patients or those with intra-abdominal disease. The presence of *APC* germline mutations and symptomatic presentation at diagnosis were associated with worse outcomes in univariate analysis, underscoring the influence of genetic predisposition (e.g., FAP) on disease behavior. While *CTNNB1* mutation status—particularly *T41A* versus *S45* variants—did not significantly correlate with EFS in our study, the sample size may have limited power in this analysis.

The role of AS is gradually gaining more importance in the management of DT [18,19]. Our series showed a rate of disease control and spontaneous regression similar to what has been previously described, with more than half of the patients who underwent an initial surveillance strategy not requiring further treatments. Of note, a significant proportion of patients who had progressed or relapsed after previous treatments also underwent AS and did not receive further treatments.

HT, frequently administered in combination with NSAIDs, has been a common treatment approach and thus has a strong representation in our cohort. However, current guidelines discourage its use due to the lack of demonstrated benefit in prospective studies [7]. For this reason, we analyzed this group of patients separately from those receiving CT or TKIs and compared them to patients undergoing AS to more accurately assess the true efficacy of active treatment strategies.

Surgical treatment, either primary (49.5%) or secondary (12%), remained a cornerstone of treatment in our cohort, particularly for anatomical sites amenable to resection. However, our findings align with the previous literature suggesting a relatively high recurrence rate post-surgery (36%), especially among externally operated patients [20,21,22]. In our series, the margin status emerged as a significant factor: R1/R2 resections were independently associated with higher event risk (HR 2.26, *p* = 0.006), as observed in other studies [23]. It is worth noting that most relapses occurred in patients referred from other institutions after previous surgery, which may have contributed to an overestimation of the recurrence rate in our cohort. This mirrors current recommendations that surgery, when employed, should aim for negative margins whenever feasible, though morbidity must be weighed carefully, particularly in critical anatomical locations.

Notably, responses to CT or TKI are active and effective with a tolerable safety profile, as reported in other series [9,10,11,12,13]. Systemic therapies—CT and TKIs—were reserved mainly for refractory or unresectable disease, representing only 5.5% of initial treatments; overall, 24% of patients received systemic therapy during their disease course. Response rates varied by agent, with TKIs demonstrating moderate efficacy but significant toxicity (grade 3–4 events in 38.5% for sorafenib alone). CT showed a more favorable toxicity profile, with only one grade 3 event. These findings underscore the importance of selecting and monitoring systemic treatments properly, striking a balance between efficacy and adverse effects.

Although GSIs—particularly nirogacestat—have been approved by several regulatory agencies for use in adult DTs, this is not yet the case in Spain, where access currently remains restricted to expanded-use programs [14,15]. As a result, only a few patients in our cohort initiated nirogacestat and with very limited follow-up; therefore, these cases were not included in the present analysis as no meaningful conclusions regarding activity or safety can be drawn.

RT was rarely used but appeared effective in selected radical settings; however, one case of radiation-induced sarcoma raises caution about long-term sequelae, particularly in genetically predisposed individuals. RT has historically been used in certain cases as a treatment option for DTs, particularly as a complementary treatment, as shown in our cohort [3,6,23,24,25]. Although its use has declined with the advent of alternative strategies, it could remain a viable option for select patients.

Cryoablation is a minimally invasive local therapy with a good safety record and effective local control, especially for difficult or recurrent tumors, though more research is needed to confirm its best use [26,27,28,29]. Although used in a limited number of cases in our cohort, cryoablation showed encouraging results, with a 69% response rate and durable disease control in most cases. Of note, repeat cryoablation was ineffective in two patients in our cohort. Long-term data are still needed to determine the durability of response and optimal patient selection.

EFS across the cohort was favorable, with a median of 57 months and no significant difference between initial conservative and active strategies (log-rank *p* = 0.168), supporting evidence that early intervention may not improve long-term outcomes in many DT cases. Notably, 40% of patients started with conservative management; of those, 58% remained untreated over a median of 22.5 months, confirming the growing trend towards non-interventional initial strategies in selected cases, especially in the absence of symptoms or life-threatening features. Among them, 22% showed spontaneous regression, including 5.5% with CR—aligning with reported rates of 20–30%. Furthermore, among the 12 patients who relapsed or progressed after active treatments and were subsequently managed with AS, two-thirds remained progression-free without additional intervention, underscoring the value of AS even in second- or later-line settings. These results emphasize the value of clinical patience in asymptomatic or minimally symptomatic DTs.

However, multivariate analysis identified conservative management as an independent predictor of events (HR 5.36, *p* = 0.001). This result should be interpreted in the context of the composite EFS definition, in which events frequently reflected treatment changes rather than definitive disease progression. Accordingly, while patients managed with active surveillance experienced earlier events—indicating the need for closer monitoring and timely therapeutic adjustment—this did not translate into inferior long-term EFS compared with upfront active treatment. In our series, the median time to treatment change was 4.5 months, reflecting early identification of progression and appropriate escalation of therapy when required. The efficacy of active therapies was substantiated by the fact that a significant number of patients remained progression-free following treatment escalation, particularly those managed surgically or with cryoablation. A significant proportion of patients (44.9%) experienced progression during their initial treatment approach, highlighting the unpredictable natural history of DTs and the importance of close monitoring, particularly for those managed conservatively.

### 4.1. Strengths and Limitations

This study provides one of the largest single-institution experiences with DT in a national reference center over a 10-year period, offering a comprehensive overview of real-world clinical practice in a multidisciplinary setting. The inclusion of 109 patients with long-term follow-up enhances the robustness of our findings, particularly in assessing long-term outcomes such as EFS and treatment durability. Our data reflect the evolving management paradigm of DTs, incorporating both conservative and active strategies, including emerging therapies such as cryoablation. By analyzing HT ± NSAIDs as a distinct conservative approach, we differentiated these patients from those receiving CT or TKIs, in contrast to prior studies that grouped both under systemic therapy. The availability of detailed clinical, pathological, radiological, and molecular data in most patients further strengthens the validity of our conclusions.

The retrospective design, however, inherently limits causal inference and introduces potential selection bias. Management strategies were not randomized and were influenced by temporal trends, physician preference, tumor location, and symptom burden. It should be noted that the treatment paradigm has evolved considerably in recent years; our cohort includes patients treated with possibly outdated strategies such as HT ± NSAIDs, which may not reflect current practices. In addition, our cohort may be biased toward patients with more aggressive disease, given that many were referred from other institutions, sometimes following suboptimal initial management.

Our study did not identify tumor location as an independent predictive factor. This may be explained by the fact that DTs were not categorized according to current evidence that suggests certain anatomical sites are associated with favorable or unfavorable outcomes [3,30]. Similarly, we did not observe the prognostic impact of certain *CTNNB1* mutations reported in prior studies, possibly due to the limited number of patients who underwent molecular testing, which restricts the statistical power of this analysis [30,31].

We also failed to quantify symptomatic burden or the need for supportive care, which are increasingly recognized as key factors in treatment selection for DT patients.

Finally, the definition of “events” in this study encompasses diverse clinical outcomes, including disease progression, relapse, and changes in therapeutic strategy. While this approach reflects real-world clinical decision-making, it may limit direct comparability with other studies that rely on more uniform endpoints such as EFS. Moreover, this composite definition may partly conflate true biological progression with treatment modifications driven by physician judgment, patient anxiety, or other non-clinical considerations. In addition, systemic therapies were heterogeneous and administered across different lines of treatment, which precluded meaningful head-to-head comparisons of efficacy among individual agents.

### 4.2. Clinical Implications and Future Directions

Our findings underscore the necessity of individualized, multidisciplinary management for patients with DTs, emphasizing a personalized, risk-adapted approach guided by multidisciplinary expertise. The substantial proportion of patients managed successfully with AS supports its role as the preferred initial strategy, especially in asymptomatic or minimally symptomatic cases. Recognizing the appropriate timing for treatment initiation is essential, particularly in anatomically critical regions or symptomatic tumors, to minimize morbidity.

Nevertheless, many patients will require active interventions. The heterogeneity of systemic therapies used and the variability in response highlight the urgent need for standardized treatment algorithms. Integration of molecular profiling, particularly *CTNNB1* mutation status, may help stratify patients according to recurrence risk and treatment sensitivity, although further validation is needed.

Due to the morbidity associated with intensive treatments and the long-lasting nature of this disease, shared decision-making and patient-tailored care that prioritize quality of life are essential.

Future directions should focus on refining prognostic models through molecular risk—including broader genetic testing—and prospective evaluation of newer systemic and locoregional therapies, evaluating patient-reported outcomes to guide treatment decisions and optimal sequencing. National and international collaborative registries, along with prospective observational and interventional studies, are critical to strengthening our understanding of the disease.

## 5. Conclusions

In conclusion, our experience supports an individualized, patient-centered approach to DT, prioritizing AS for low-risk disease, timely escalation upon progression, careful surgical planning to obtain clear margins when intervention is required, along with judicious use of systemic or ablative therapies. Given that DT remains a rare but clinically impactful disease, centralization of care within specialized referral networks is essential to optimizing outcomes and ensuring equitable access to emerging treatments and clinical trials.

## Figures and Tables

**Table 1:** Baseline characteristics by initial treatment strategy (conservative vs. active treatment).

		Global Cohort *n* = 109	Conservative Therapy *n* = 44 (AS 24/HT ± NSAIDs 20)	Active Therapy *n* = 65 (CT/TKIs *n* = 6, Surgery *n* = 54, Cryoablation *n* = 4, RT *n* = 1)
Age, median (IQR)		36.8 (29–48)	37 (31.3–45.8)	36.1 (26.5–50.3)
Female, *n* (%)		62 (56.9)	25 (56.8)	37 (33.9)
Primary site, *n* (%)	Abdominal wall	22 (20.2)	14 (31.8)	8 (7.3)
	Intra-abdominal	35 (32.1)	12 (27.3)	23 (21.1)
	Head & Neck/Intrathoracic	15 (13.8)	8 (18.2)	7 (6.4)
	Extremity/girdles/chest wall	37 (33.9)	10 (22.7)	27 (24.8)
Multifocal, *n* (%)		24 (22.0)	17 (38.6)	7 (6.4)
Tumor size ≥ 5 cm, *n*/N ^a^ (%)		57/103 (55.3)	19/42 (45.2)	38/61 (62.3)
Germline *APC* mutation, *n*/N ^a^ (%)		18/40 (45)	10/20 (50.0)	8/20 (40.0)
Prior local surgery, *n* (%)		14 (12.8)	16 (36.4)	8 (12.3)
Symptoms at diagnosis, *n* (%)		52 (47.7)	20 (45.5)	32 (49.2)

^a^. *N* is the number of assessable patients for the variable. AS: Active surveillance. HT: Hormone therapy. NSAIDs: non-steroidal anti-inflammatories. CT: Chemotherapy. TKIs: tyrosine-kinase inhibitors. RT: Radiotherapy. IQR: Interquartile range.

**Table 2:** Frequencies of *CTNNB1* mutation subtypes.

		**Molecular Study** **Conducted *N* = 29 (%)**	**Global Cohort** ***N* = 109 (%)**
*CTNNB1* mutation (*n* = 29)	*T41A*	17 (58.6)	15.6
	*S45P*	3 (10.3)	2.75
	*S45F*	3 (10.3)	2.75
	Any mutation	23 (79.3)	21.1

Molecular analysis was available for 29 patients. Percentages in the global cohort column (*N* = 109) are calculated using the entire study population as the denominator and therefore reflect the minimum known prevalence of each mutation.

**Table 3:** Patterns of treatment sequencing and modality use across tumor sites.

Treatment Modalities Received During Disease Course ^a^		AS *n* = 36 (33%) ^b^	HT ± NSAIDs *n* = 32 (29.3%)	Anti-Cancer Drugs *n* = 26 (23.9%)	Surgery *n* = 67 (61.4%)	Cryoablation *n* = 13 (11.9%)	RT *n* = 10 (9.2%)
Line of treatment (*n*, %)	1st line	24 (66.7)	20 (62.5)	6 (23.1)	54 (80.6)	4 (30.8)	1 (10)
	2nd line	7 (19.4)	8 (25)	8 (30.8)	11 (16.4)	6 (46.2)	1 (10)
	>3rd line	5 (13.9)	-	11 (42.3)	2 (2.9)	3 (23.1)	2 (20)
	Perioperative ^c^	-	4 (12.5)	1 (3.8)			6 (60)
Primary site (*n*, %)	Abdominal wall	9 (25.0)	5 (15.6)	4 (15.4)	12 (17.9)	3 (23.1)	1 (10)
	Intra-abdominal	14 (38.9)	12 (37.5)	9 (34.6)	24 (35.8)	0	1 (10)
	Head & Neck/Intrathoracic	0	1 (3.1)	3 (7.7)	5 (7.5)	1 (7.7)	2 (20)
	Extremity/girdles/chest wall	13 (36.1)	14 (43.8)	11 (42.3)	26 (38.8)	9 (69.2)	6 (60)
	Multifocal	7 (19.4)	13 (40.6)	10 (38.5)	11 (16.4)	2 (15.4)	3 (30)

Abbreviations: AS, active surveillance; RT, radiotherapy; NSAIDs, non-steroidal anti-inflammatory drugs; HT, hormonal therapy. ^a^ More than one treatment modality could be received per patient. ^b^. Percentages are calculated over the total number of patients receiving each modality. ^c^. Perioperative radiotherapy included adjuvant (*n* = 4) and intraoperative (*n* = 2) intent.

**Table 4:** Distribution and outcomes of the most frequent anti-cancer drugs.

Regimen	*n*	1st Line (*n*)	≥2nd line (*n*)	ORR (%, 95% CI)	PFS (Months, 95% CI)	Median Follow-Up (Months, IQR)
**Anthracycline-based ^a^**	16	9	7	42.9 (16.9–68.8)	18 (9.5–26.4)	24 (18–42)
**Methotrexate–vinblastine**	8	2	6	50 (15.3–84.7)	7.9 (4.9–29)	18 (14–35)
**Sorafenib**	13	8	5	38.5 (13.9–68.4)	9 (4–23)	23 (10–26)

^a^ All patients received pegylated liposomal doxorubicin except for two cases receiving the combination of doxorubicin-dacarbazine.

## Data Availability

The datasets presented in this article are not publicly available because they are part of an internal hospital database with restricted access and can only be accessed by members of the multidisciplinary sarcoma team at Hospital General Universitario Gregorio Marañón. Requests to access the datasets or further inquiries can be directed to the corresponding author.

## Abbreviations

AS	active surveillance
APC	adenomatous polyposis coli
BRCA1	breast cancer gene 1
CI	confidence interval
CR	complete response
CT	chemotherapy
CT scan	computed tomography
CTNNB1	catenin beta 1
DT	desmoid tumor
DTs	desmoid tumors
EFS	event-free survival
FAP	familial adenomatous polyposis
GCIs	Gamma-secretase inhibitors
HR	hazard ratio
HT	hormonal therapy
MDT	multidisciplinary team
MRI	magnetic resonance imaging
MUTYH	mutY DNA glycosylase
NSAIDs	non-steroidal anti-inflammatory drugs
ORR	overall response rate
OS	overall survival
PD	Progressive disease
PFS	progression-free survival
PR	partial response
POT1	protection of telomeres 1
RT	Radiotherapy
RECIST	Response Evaluation Criteria in Solid Tumors
SD	stable disease
TKIs	tyrosine kinase inhibitors
WHO	World Health Organization
